# *Pleurolucina* from the western Atlantic and eastern Pacific Oceans: a new intertidal species from Curaçao with unusual shell microstructure (Mollusca, Bivalvia, Lucinidae)

**DOI:** 10.3897/zookeys.620.9569

**Published:** 2016-09-29

**Authors:** Emily A. Glover, John D. Taylor

**Affiliations:** 1 Department of Life Sciences, The Natural History Museum, London SW7 5BD, UK

**Keywords:** Bacterial symbionts, Caribbean, conchiolin layers, defensive adaptation, Lucinidae, Pleurolucina

## Abstract

A new shallow water species of the lucinid bivalve *Pleurolucina* is described from Curaçao in the southern Caribbean Sea and compared with known species of the genus from the western Atlantic and eastern Pacific Oceans. Although confused with the Floridian species *Pleurolucina leucocyma*, it is most similar to the eastern Pacific *Pleurolucina undata*. As in all studied lucinids, the new species possesses symbiotic bacteria housed in the ctenidia. The shell microstructure is unusual with repeated and intercalated conchiolin layers that have sublayers of ‘tulip-shaped’ calcareous spherules. Predatory drillings by naticid gastropods frequently terminate at the conchiolin layers.

## Introduction

The tropical and subtropical western Atlantic is one of the major centres of marine molluscan diversity and bivalves in the speciose family Lucinidae, with an estimated 46 species in this ocean, have been the focus of many studies since the discovery of their chemosymbiosis with sulphide-oxidising bacteria (e.g. Giere 1985, Fisher and Hand 1984, Frenkiel and Mouëza 1995, Frenkiel et al. 1996, Gros et al. 1998, 1998, 2012). Nonetheless, new species from both shallow and deep water are still discovered and new genera identified (Taylor and Glover 2009, Taylor et al. 2013). Additionally, within the area there are several cryptic species with narrower ranges nestled among supposedly widespread species (Huber 2015, Taylor and Glover submitted. Distributional data for western Atlantic lucinids indicates that although some are widespread, others have more restricted ranges. A recurring pattern is of congeneric pairs, one largely restricted to the Gulf of Mexico and Florida and the other with a more southerly Caribbean range as exemplified by *Lucinisca nassula* and *Lucinisca muricata* (Taylor and Glover submitted). This dual distribution is similar to that proposed by Petuch (1982) as a relict of the Caloosahatchee-Gatunian pattern dating from the Pliocene but possibly inherited by present day taxa. Additionally, in the eastern Pacific, there are lucinids closely similar morphologically and genetically to those of the western Atlantic and presumably separated by the rise of the Central American Isthmus around 3.5 mya. Examples of these are the pair *Radiolucina amianta* (Atlantic) and *Radiolucina cancellaris* (Pacific) (see Garfinkle 2012), and the pair *Ctena imbricatula* (Atlantic) and *Ctena mexicana* (Pacific) (Taylor et al. 2011).

*Pleurolucina* (Dall, 1901) is a genus of small lucinids characterised by broad radial ribs. The type species, *Lucina leucocyma* Dall, 1886, first described from off the Florida Keys, is documented as having a distribution from North Carolina to Colombia including Yucatan Peninsula (Britton 1970, Vokes and Vokes 1983, Huber 2015). Two other species, *Pleurolucina hendersoni* Britton, 1972 and *Pleurolucina sombrerensis* (Dall, 1886), are known from the western Atlantic (Britton 1972), while three further species are recorded from the Eastern Pacific (Coan and Valentich-Scott 2012). During field sampling in shallow seagrass around Curaçao in May 2015 we collected a *Pleurolucina* that we recognised as similar to, but likely distinct from, *Pleurolucina leucocyma*. Further research showed this to be an undescribed species more widely distributed in the southern Caribbean and confounded with *Pleurolucina leucocyma*. An apparent high incidence of failed naticid drill holes focused attention on the shell microstructure revealing intercalated organic layers. Thought to be related to *Lucina* or *Cavilinga* (Britton 1972, Bretsky 1976) and included by Taylor et al. (2011) in the subfamily Lucininae, no *Pleurolucina* species has previously been included in molecular analyses.

We describe this new *Pleurolucina* from Curaçao in comparison with other western Atlantic and Eastern Pacific species, detail its phylogenetic position and illustrate its unusual shell microstructure with calcified conchiolin layers.

## Material and methods

Samples of the new species were collected in southern Curaçao – location below. Details of ctenidia and sperm were studied using critical point dried glutaraldehyde-fixed specimens. Shells, microstructure and anatomy were imaged using a Quanta FEI 650 FEG scanning electron microscope. Comparative shell material was studied in USNM and NHMUK.

**Institutional abbreviations**

FMNH
Field Museum of Natural History, Chicago, USA 


MCZ
Museum of Comparative Zoology, Harvard University, USA 


MNHN
Muséum national d’Histoire Naturelle, Paris, France 


RMNH
 Rijksmuseum van Natuurlijke Histoire, Leiden, Netherlands 


NHMUK
 The Natural History Museum, London, UK 


SBMNH
Santa Barbara Museum of Natural History, USA 


USNM
 United States National Museum of Natural History, USA 


**Other abbreviations**

H shell height


L shell length


LV left valve


PI protoconch I length


PII protoconch II length


RV right valve


SEM scanning electron microscopy


T tumidity single valve


## Systematics

### Family Lucinidae Fleming, 1828 Subfamily Lucininae Fleming, 1828

#### Pleurolucina

Taxon classificationAnimaliaLucinidaLucinidae

Dall, 1901

Dallucina Olsson & Harbison, 1953. Type species, by original designation, Lucina (Here) amabilis Dall, 1898. Pliocene, Florida. Gender feminine.

##### Type species.

*Lucina leucocyma* Dall, 1886, by original designation. Recent, western Atlantic Ocean. Gender feminine.

##### Diagnosis.

Shell small, L to 27 mm (*Pleurolucina sombrerensis* usually less than 10 mm), subcircular to ovate, generally higher than long, inflated to highly inflated. Sculpture of 4–6 broad radial ribs separated by broad sulci, sometimes absent in adult shells, crossed by closely-spaced, often terraced, commarginal lamellae. Lunule deeply excavated to shallow. Ventral margin finely beaded. Hinge: RV with two cardinal teeth, posterior-most sometimes bifid, anterior and posterior lateral teeth present; LV with two cardinal teeth, anterior smaller, with anterior and posterior lateral teeth. Anterior adductor muscle scar relatively short, broad, separate from pallial line for about ½ to 2/3 of length, pallial line entire.

##### Included species.

Western Atlantic: *Pleurolucina leucocyma* (Dall, 1886), *Pleurolucina hendersoni* Britton, 1972, *Pleurolucina sombrerensis* (Dall, 1886). Eastern Pacific: *Pleurolucina leucocymoides* (Lowe, 1935), *Pleurolucina taylori* Coan & Valentich-Scott, 2012, *Pleurolucina undata* (Carpenter, 1865).

##### Distribution.

Western Atlantic: northern Florida to Brazil (*Pleurolucina sombrerensis* Espirito Santo, Rios 1994). East Pacific: Baja California Mexico to Ecuador, Galapagos Islands (Coan and Valentich-Scott 2012).

##### Geological range.

Early Oligocene to Recent. *Pleurolucina amabilis* (Dall, 1898) is a distinctive, laterally compressed species from the Late Pliocene to mid-Pleistocene of Florida. It was made type species of the new genus *Dallucina* by Olsson and Harbison (1954) but other than the lateral compression it is similar in most characters to *Pleurolucina leucocyma*. From Miocene deposits of Ecuador Olsson (1964) described *Paslucina* with Lucina (Paslucina) follis Olsson, 1964 as type species. This has the shape and radial folds typical of *Pleurolucina* species and may be an antecedent.

*Pleurolucina quadricostata* (Dall, 1903) from the Pliocene Bowden Formation of Jamaica (Woodring 1925: 121, pl. 16, figs 4-6) resembles the living *Pleurolucina leucocyma*. From the same deposit, Phacoides (Linga) tithonis (Dall, 1903) (Woodring 1925: 120, pl. 16, figs 2, 3) is similar to *Pleurolucina sombrerensis*. A species described as Lucina (Cavilinga) triloba (Dockery 1982, pl. 19, fig 4) from the Early Oligocene, Vicksburg Group, Mississippi, USA, has characters of *Pleurolucina* but with only two radial folds. From the same deposits, Lucina (Cavilinga) imbricolamella Dockery (1982 pl. 20, figs 11–12) resembles the Recent *Pleurolucina sombrerensis*.

##### Relationships.

From morphological characters of the shells, *Pleurolucina* species are usually regarded as being related to *Lucina* s.s. or *Cavilinga* (Britton 1972, Bretsky 1976). *Pleurolucina harperae* below is the only member of the genus yet to be included in molecular analyses and results (Taylor et al. submitted) show that it groups within the Lucininae, close to *Cavilinga blanda*, in a subclade of *Lucina* and *Divalinga* species.

##### Remarks.

In the absence of molecular evidence, other than for *Pleurolucina harperae*, our concept of *Pleurolucina* embraces a range of shell morphologies from species like *Pleurolucina leucocyma*, *Pleurolucina undata* and *Pleurolucina taylori* that have prominent radial ribs, through the less ribbed *Pleurolucina hendersoni* and *Pleurolucina leucocymoides*, to the small *Pleurolucina sombrerenis* that has a rounded shell lacking radial ribs. Nevertheless, they are all rather inflated with similar dentition, anterior adductor muscle scars and beaded inner margins.

#### Pleurolucina harperae

Taxon classificationAnimaliaLucinidaLucinidae

n. sp.

http://zoobank.org/D9916BAC-D208-4A5B-8499-6FE1B5ADC3BB

**Figure 1.**
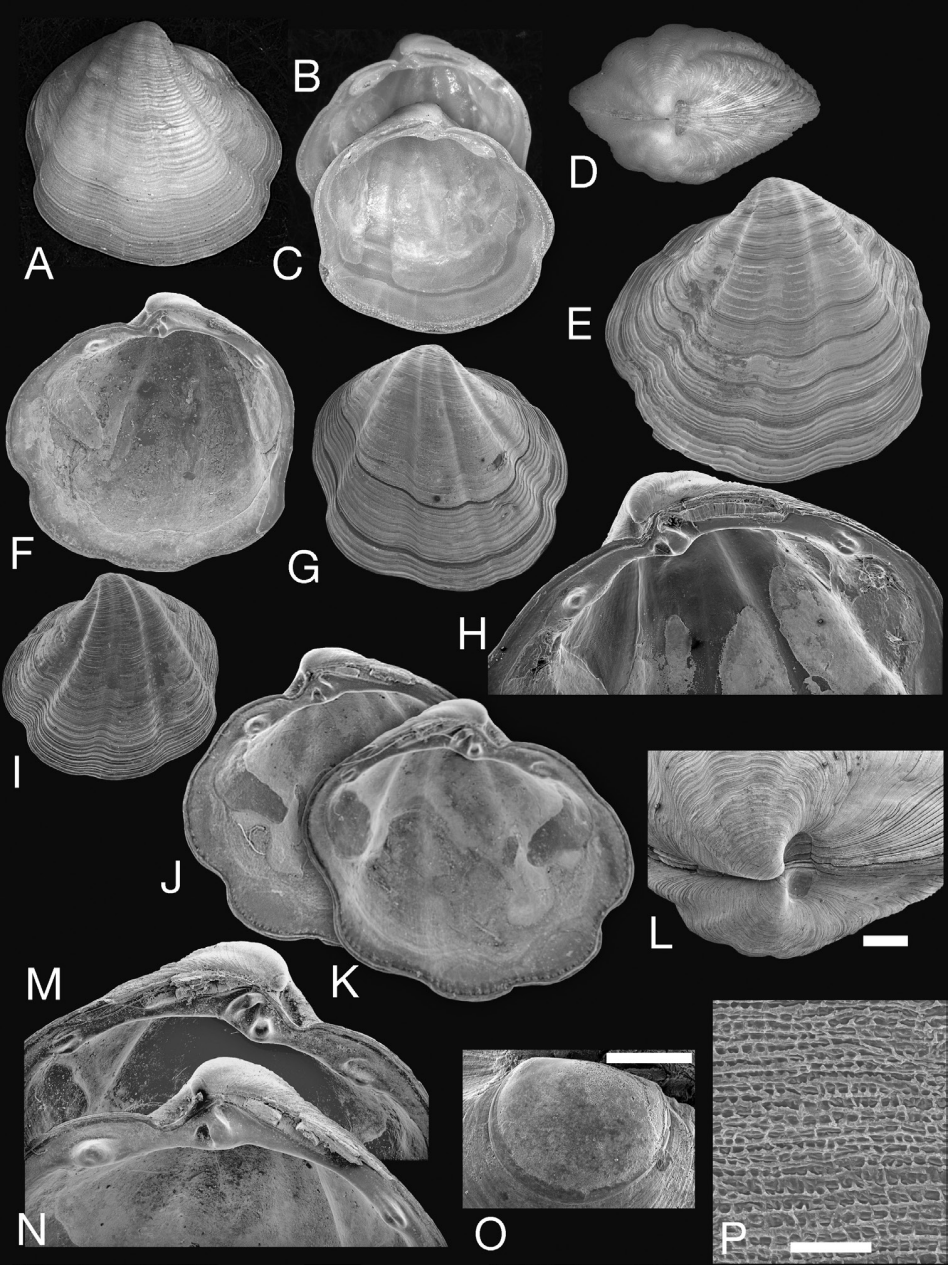
*Pleurolucina harperae* sp. n. **A–C** Holotype NHMUK 20160338 exterior of right and interior of right and left valves L 8.8 mm. **D–P** Paratypes. NHMUK 20160339 dorsal view L 7.6 mm. **E** Exterior of left valve L 7.7 mm. **F** Interior of right valve L 6.3 mm. **G** Exterior of right valve L 7.9 mm. **H** Hinge area of right valve L 8.6 mm. **I** Exterior of left valve L 63 mm. **J, K** Interiors of right and left valves L 5.0 mm. **L** Dorsal view showing lunule. Scale bar = 0.5 mm. **M, N** Details of hinge teeth of J, K. **O** Protoconch. Scale bar = 100 µm. **P** Detail of microsculpture. Scale bar = 20 µm.

**Figure 2.**
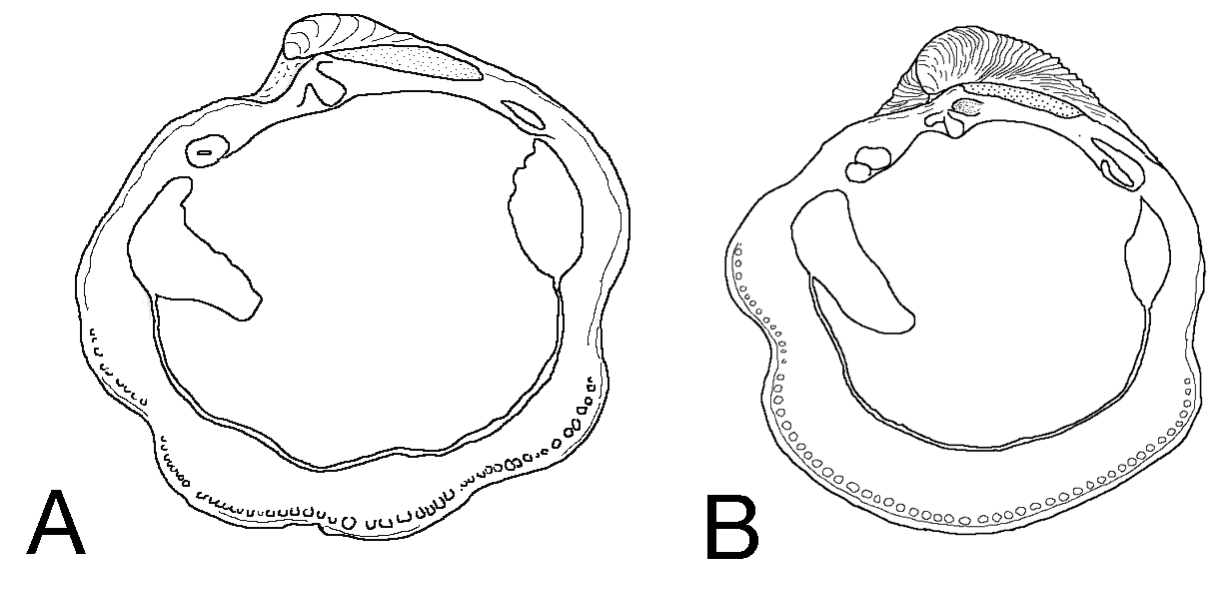
Outline drawings of shell interiors of **A** *Pleurolucina harperae* and **B** *Pleurolucina leucocyma*.

**Figure 3.**
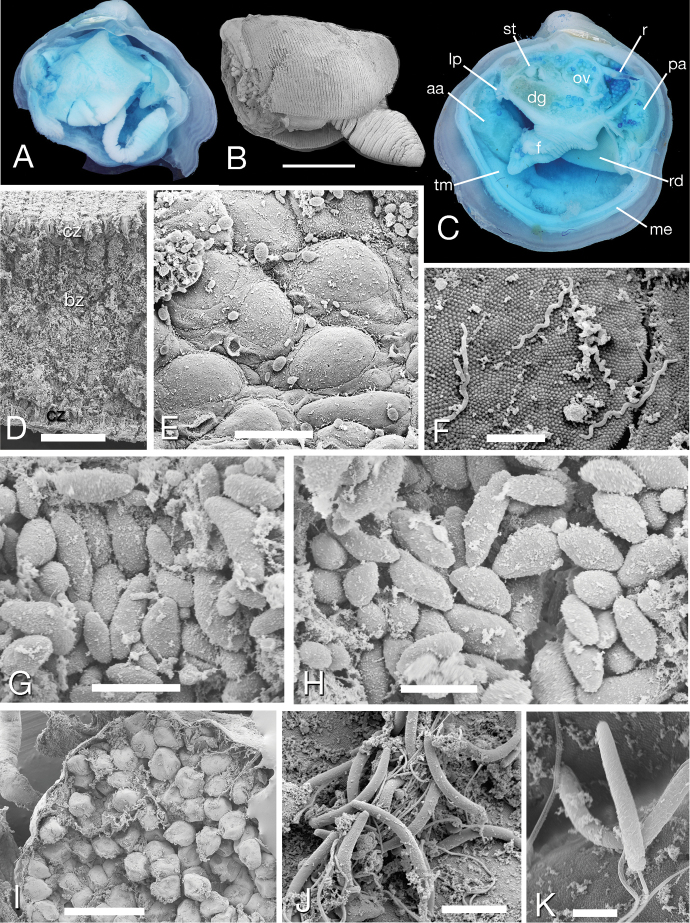
*Pleurolucina harperae*, general anatomy, ctenidia, bacteria, oocytes and sperm. **A** Right side, with mantle removed, right demibranch and extended foot stained with methylene blue L 7 mm **B** Left demibranch and foot, critical point dried preparation. Scale bar = 1 mm **C** Cut section to show general anatomy, stained with methylene blue L 8 mm **D** Transverse section through single ctenidial demibranch. Scale bar = 100 µm **E** Surface of bacteriocytes and intercalary cells on lateral view of a ctenidial filament. Scale bar = 15 µm **F** Spirochaete bacteria on surface of bacteriocytes. Scale bar = 2 µm **G, H** Symbiotic bacteria contained in bacteriocyte. Scale bar = 5 µm **I** Developing oocytes. Scale bar = 500 µm **J, K** Sperm. Scale bars = 5, 2 µm respectively. **aa** anterior adductor muscle **bz** bacteriocyte zone **cz** ciliated zone **dg** digestive gland **f** foot **lp** labial palps **me** mantle edge **ov** ovary with oocyctes **pa** posterior adductor **r** rectum **rd** right demibranch **st** stomach **tm** thickened mantle ventral to anterior adductor muscle.

**Figure 4.**
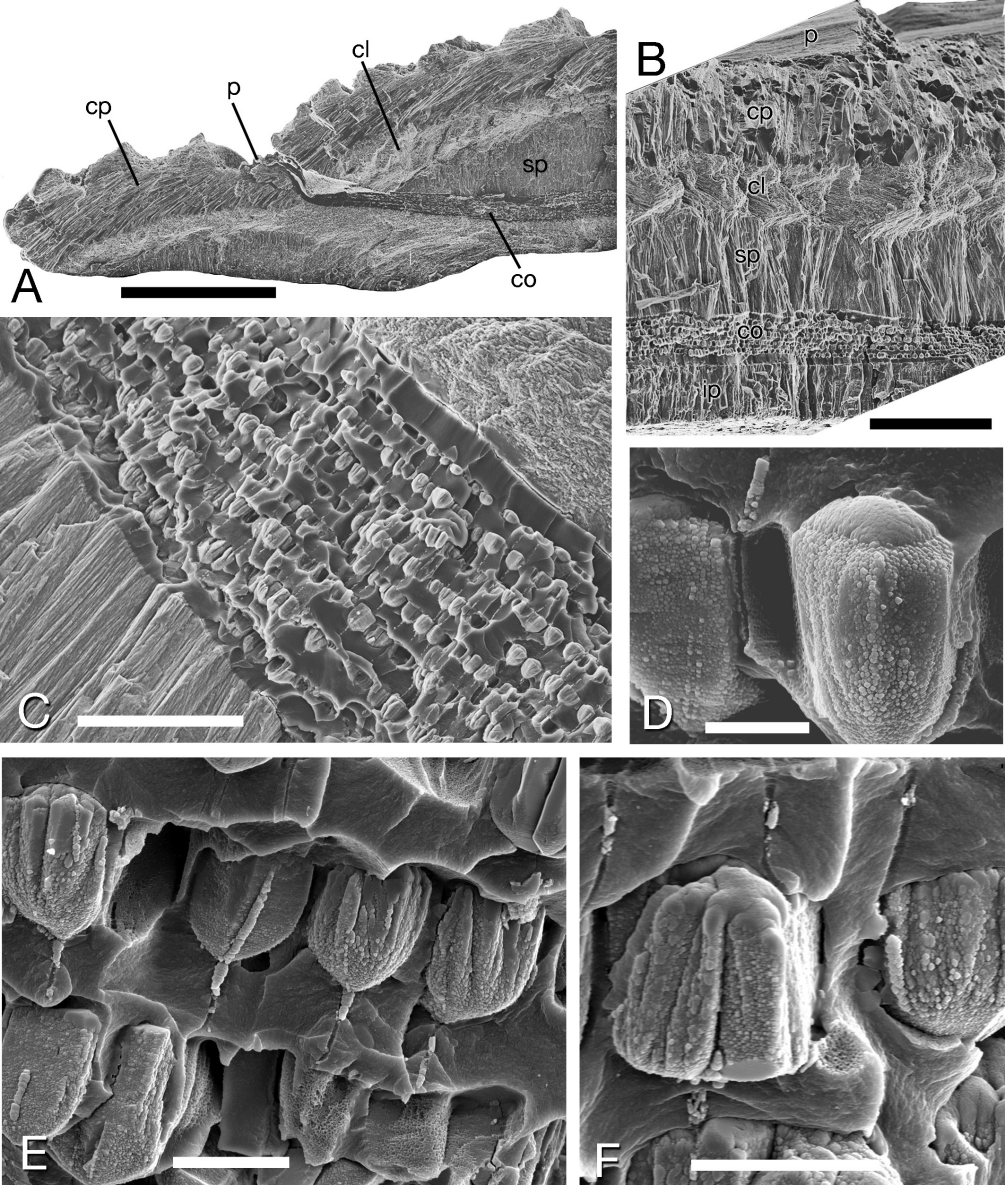
Shell microstructure of *Pleurolucina harperae*. **A** Fractured section of shell margin showing major notch growth halt and conchiolin layer. Scale bar = 400 µm **B** Fractured section showing succession of shell layers. Shell exterior at top. Scale bar = 100 µm **C** Conchiolin layer with regular bands of spherulites. Scale bar = 40 µm **D** Individual spherulite. Scale bar = 2 µm **E** Adjacent spherulites embedded in conchiolin with narrow channels between layers. Scale bar = 5 µm **F** Single spherulites with channels below and above. Scale bar = 5 µm. **cl** crossed lamellar layer **co** conchiolin layer **cp** composite prismatic layer **ip** irregular prismatic layer **p** periostracum **sp** spherulitic prismatic layer.

**Figure 5.**
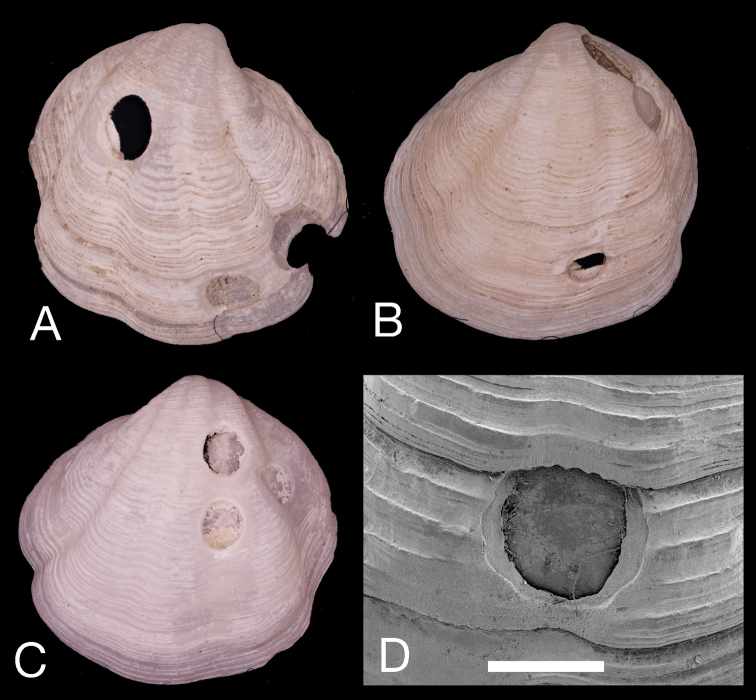
Failed and multiple drill holes in shells of *Pleurolucina harperae*. **A** L = 6.8 mm **B** L = 9.8 mm **C** L =7.8 mm **D** SEM of failed drill hole terminating at conchiolin layer. Scale bar = 1.0 mm.

Figs 1 , 2 , 3 , 4 , 5

Lucina leucocyma : Daccarett and Bossio 2011: 177, fig. 1243.
Pleurolucina leucocyma : Huber 2015: 433, fig. p. 85.

##### Type material.

*Holotype*: 1 whole shell L 8.8, H 8.5 T 3.2 mm (NHMUK 20160338), southwestern Curaçao, channel into Spaanse Water, opposite Hyatt Resort, 12°03’57” N 68°51’13” W. BivAToL stn Cur-5-15-009, 22 May 2015.

*Paratypes*: 92 valves (NHMUK 20160339), 2 paired valves (RMNH 5003991–50003992), 3 paired valves (FMNH344698), 2 paired valves (USNM 1411553). Same locality as holotype.

##### Other material.

19 ethanol preserved specimens (NHMUK), same locality as holotype.

##### Description.

Shell subovate, slightly anteriorly extended, L to 9.6 mm, H to 9.7 mm, H/L 0.99, moderately inflated, sculpture of flat, closely spaced commarginal lamellae, with four prominent, broad ribs with interspaces variable in width, but always narrower than ribs themselves; microsculpture of tight rows of shallow pits (Fig. 1 P). Umbones low, situated on midline. Anterior dorsal area arcuate. Protoconch: PI 217 µm, PI + PII 228 µm, PII a narrow rim with fine increments (Fig. 1 O). Lunule short, semicircular, slightly impressed. Ligament short, set in shallow resilifer. Hinge teeth: LV with two cardinal teeth; a robust anterior lateral tooth and smaller posterior lateral. RV with a single large cardinal tooth and anterior and posterior lateral teeth. Anterior adductor muscle scar short, broad, widely divergent from pallial line (60–70 µm) for about half of length (Fig. 2 A), posterior scar ovate; pallial line entire, pallial blood vessel scar sometimes visible. Shell margin finely beaded, sinuate with anterior sinus deeper. Shell within pallial line often patchily eroded to expose inner shell layers. Colour grey-white.

##### Anatomy.

General anatomy resembles most other described lucinids (Fig. 3). Mantle fusion ventral to the posterior apertures is very short. Foot short and broad when retracted but can be vermiform when extended (Fig. 3 A) with a small heel. Visceral pouches absent. Distinct mantle gills are absent but the inner mantle ventral to the anterior adductor muscle is thickened (Fig. 3 C) and may be a respiratory area with blood space as seen in other lucinids (Taylor and Glover 2000). Labial palps are very short. In common with all other studied Lucinidae, *Pleurolucina harperae* has ctenidia comprising inner demibranchs only; these were pink in life, large, thick and occupying much of the mantle cavity (Fig. 3 B). Ctenidial filaments are approx. 40 µm thick and 380 µm deep with a narrow 45 µm ciliated zone and a deep bacteriocyte zone (Fig. 3 D). Bacteriocytes were packed with ‘potato-shaped’ bacteria 3–5 µm long and 1.5–2.0 µm wide (Figs 3 G, H). The surface of the microvilli-covered bacteriocytes and intercalary cells were colonised by abundant spirochaetes 2.5 µm long and 0.2 µm wide (Fig. 3 F) similar to those reported by Ball et al. (2009) from *Euanodontia ovum* (Reeve, 1850). In comparison the symbiotic bacteria of *Clathrolucina costata* collected at the same time and same habitat were longer and rod shaped, 8–10 µm in length and approx. 1 µm wide.

The sperm of *Pleurolucina harperae* were 9 µm long and 1.2 µm wide at the base, tapering and curved distally (Figs 3 J, K). From the same locality, sperm of *Clathrolucina costata* were shorter, 4.8–5 µm and 1–1.2 µm wide with blunt tips. Oocytes of *Pleurolucina harperae* were approx. 200 µm in diameter (Fig. 3 I). Comparative sperm data is available for a few other western Atlantic lucinids (Bigatti et al. 2004); sperm of *Codakia orbicularis* were 14–15 µm long, tapering with a width of 0.8 µm; *Ctena orbiculata* were cylindrical, slightly curved, 7.5 µm long and 1–1.2 µm wide at base and *Lucina pensylvanica* were 15.5 µm long, with curved tapering heads and 1.1 µm wide at the posterior.

##### Shell microstructure.

Within a very thin (ca 1 µm) periostracum, *Pleurolucina harperae* has a basic four layered shell (Figs 4 A,B); an outer composite prismatic layer, followed inwards by a thin crossed-lamellar layer, then a thicker layer of irregular spherulitic prisms and within the pallial line a complex crossed-lamellar layer with sublayers of irregular prisms. The shell layers are interrupted by sheets of conchiolin around 20–90 µm in thickness, each with repeated sublayers of small discrete ‘tulip-shaped’ calcified spherulites approx. 5 µm in diameter (Figs 4 D, F). Each spherulite is joined to those of the layer below with a narrow (0.5 µm) semicalcified channel through the conchiolin (Figs 4 E, F). At the shell surface, the conchiolin sheets correspond to major depositional halts (Fig. 4 A) visible as notches in the shell with the conchiolin appearing contiguous with the invaginated periostracum. In each shell there may be between 1–5 of such sheets.

Drill holes in *Pleurolucina harperae* produced by predatory naticid gastropods were observed with full penetration in 14 out of 114 single valves, but with 12 records of incomplete drill holes that terminated at an internal conchiolin layer (Fig. 5). In one shell there were three failed drills and in another two failures before successful penetration. Incidences of apparent multiple completed drill holes in dead shells may have resulted from post-mortem degradation of organic layers in failed drill holes.

**Figure 6.**
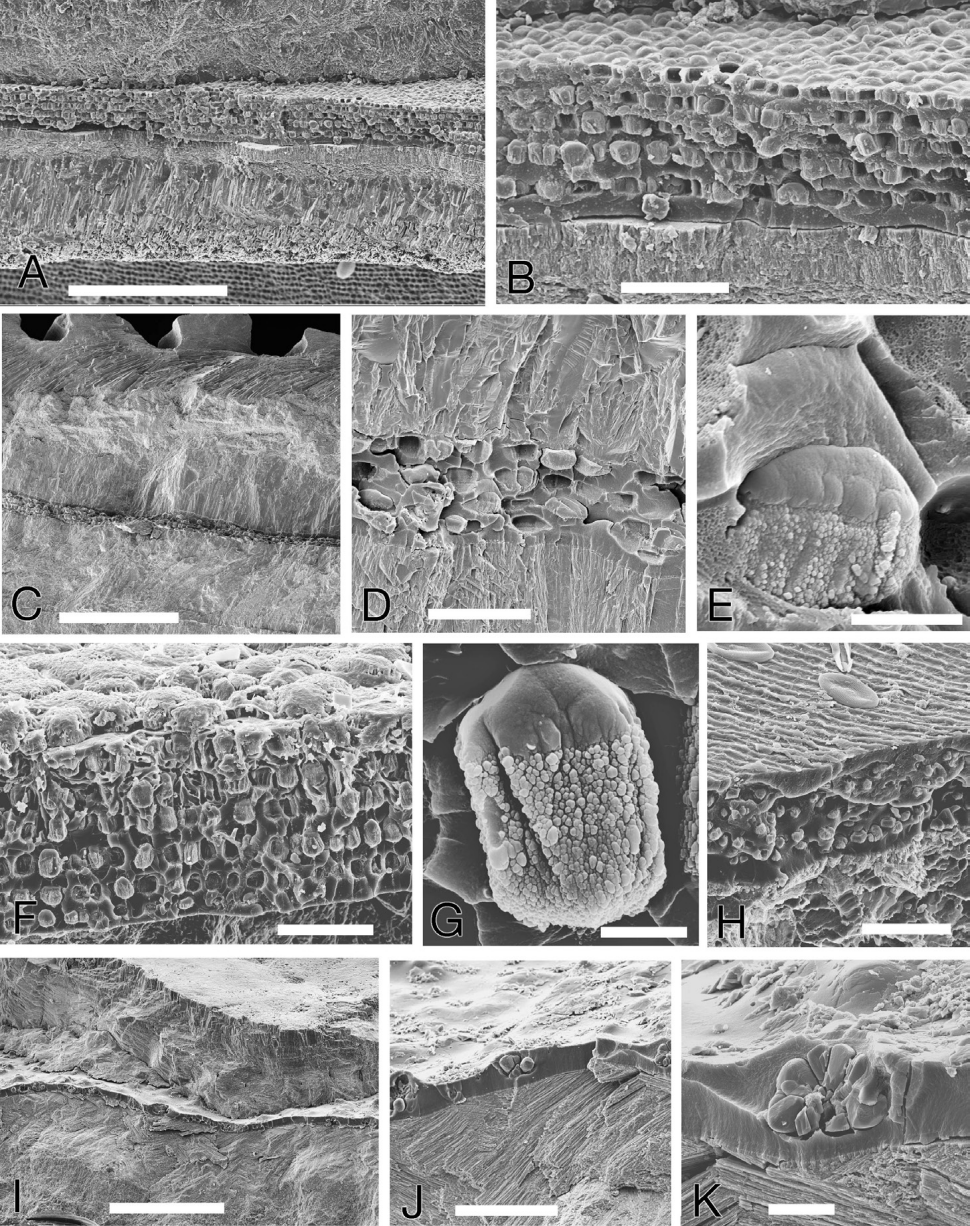
Shell microstructure of other species *Pleurolucina hendersoni*, *Pleurolucina undata*, *Lucina pensylvanica* and *Cardiolucina quadrata*. **A** *Pleurolucina hendersoni* Guadeloupe, fractured section with prominent calcified conchiolin layer, periostracum at base. Scale bar = 20 µm **B** *Pleurolucina hendersoni*, detail of conchiolin layer with lines of calcareous spherulites. Scale bar = 20 µm **C** *Pleurolucina undata* Baja California, fractured section with thin conchiolin layer Scale bar = 200 µm **D** *Pleurolucina undata*, detail of conchiolin layer with spherulites. Scale bar = 20 µm **E** *Pleurolucina undata*, single spherulites embedded in conchiolin. Scale bar = 3 µm **F** *Lucina pensylvanica* Florida Keys, calcified conchiolin layer. Scale bar = 20 µm **G** *Lucina pensylvanica*, single spherulite. Scale bar = 2 µm **H** *Lucina pensylvanica*, section of periostracum with calcareous granules. Shell interior to top. Scale bar = 20 µm **I** *Cardiolucina quadrata* Philippines, fractured section with conchiolin layer. Scale bar = 200 µm **J** *Cardiolucina quadrata* detail of conchiolin layer with calcareous aggregates. Scale bar = 50 µm **K** *Cardiolucina quadrata* detail of calcareous aggregate. Scale bar = 10 µm.

Similar conchiolin calcified sheets were identified in *Pleurolucina hendersoni* (Figs 6 A, B) and *Pleurolucina undata* (Figs 6 C–E) but not in *Pleurolucina leucocyma* (2 shells examined) or *Pleurolucina sombrerensis* (2 shells examined). Also conchiolin sheets with multiple layers of calcareous spherules were observed in *Lucina pensylvanica* from the Florida Keys (Figs 6 F,G), apparently confined to the inner shell layer within the pallial line. This is distinct from the calcified periostracum of this species (Fig. 6 H) as described by Taylor et al. (2004). No conchiolin sheets were observed in a single *Cavilinga blanda* examined. For comparison, the repeated conchiolin sheets reported in *Cardiolucina* species by Ishikawa and Kase (2007) were studied in *Cardiolucina quadrata* from the Philippines. These sheets were approx. 10-15 µm thick and only lightly calcified with sporadic spherulitic crystal aggregations (Figs 6 I-K) with no multiple sub-layers.

##### Habitat.

*Pleurolucina harperae* is an intertidal to shallow subtidal species collected from sand amongst seagrass rhizomes (largely *Thalassia testudinum*, *Halodule* sp.) in contrast to *Pleurolucina leucocyma* that is usually recorded from deeper water, for example 30–180 m around the Florida Keys (Britton 1970). Records of *Pleurolucina harperae* from Atlantic Panama (USNM below) are also from shallow water seagrass habitats. At Curaçao it co-occurred with several other lucinid species: *Clathrolucina costata* (d’Orbigny, 1845), *Ctena imbricatula* (C.B. Adams, 1845), *Anodontia alba* Link, 1807, *Codakia orbicularis* (Linnaeus, 1758), *Lucina roquesana* J. & W. Gibson-Smith, 1982 and *Divalinga quadrisulcata* (d’Orbigny, 1845).

##### Distribution.

Southern Caribbean: Panama (USNM 759784; 620716, 759825) Colombia -Taganga (Daccarett and Bossio 2011), Curaçao. The distribution of *Pleurolucina harperae* in the southern Caribbean is uncertain but it may be restricted to the southwestern area. There have been no records from the Antilles and intensive sampling of molluscs around Guadeloupe by Muséum national d’Histoire Naturelle (KARUBENTHOS 2012, 2015) recorded only *Pleurolucina hendersoni* and *Pleurolucina sombrerensis* (Taylor and Glover submitted). Similarly, only *Pleurolucina sombrerensis* was recorded from a recent survey of the marine molluscan fauna of French Guiana (MNHN - GUYANE 2014).

##### Etymology.

Named for Elizabeth (Liz) Harper, University of Cambridge, bivalve researcher, colleague and friend, who helped collect the new species.

##### Comparison with other species.

**Figure 7.**
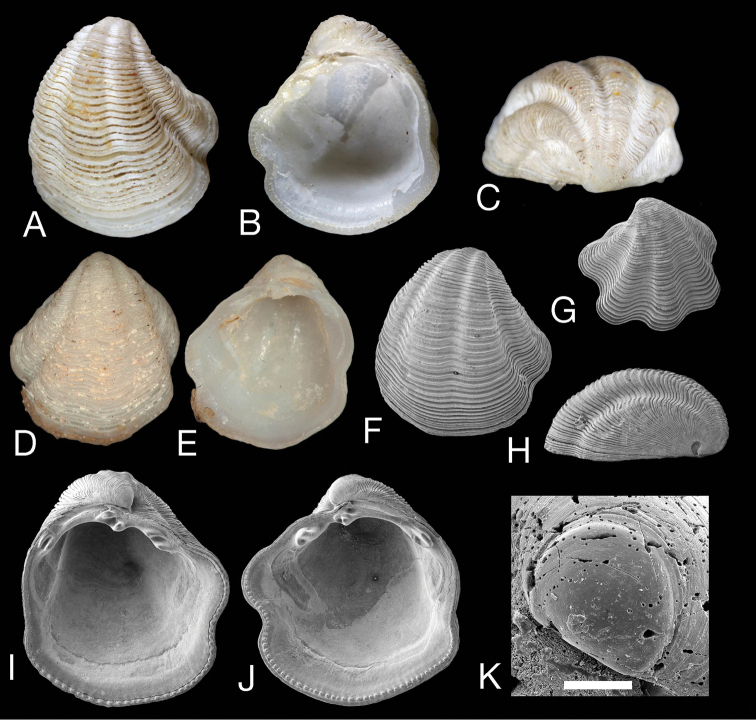
*Pleurolucina leucocyma*. **A–C** *Lucina leucocyma* Dall, 1881 lectotype MCZ 7986, exterior, interior and dorsal view of right valve, L 5.7 mm, H 6.6 mm **D, E** *Lucina leucocyma* paralectotype USNM 83140, exterior of left valve and interior of right valve, L 4.8 mm, H 5.5 mm **F–K**, *Pleurolucina leucocyma* USNM 446563 Eolis Station 368, off Ajax Reef, Florida **F** Exterior of left valve, L 5.1 mm **G** Left valve of juvenile shell, L 3.1 mm **H** Lateral view of left valve, L 5.1 mm. **I** Interior of left valve, L 5.5 mm **J** Interior of right valve, L 5.5 mm **K** Protoconch, scale bar = 100 µm.

**Figure 8.**
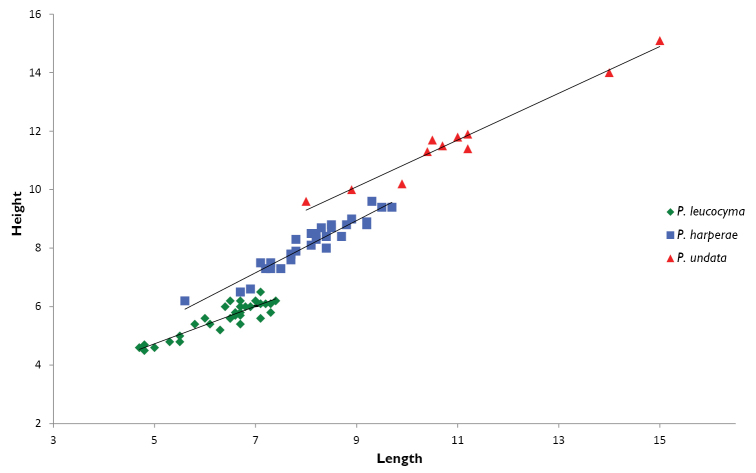
Bivariate height/length plots comparing *Pleurolucina harperae* with *Pleurolucina leucocyma*, and *Pleurolucina undata*. Length and height in millimetres.

**Figure 9.**
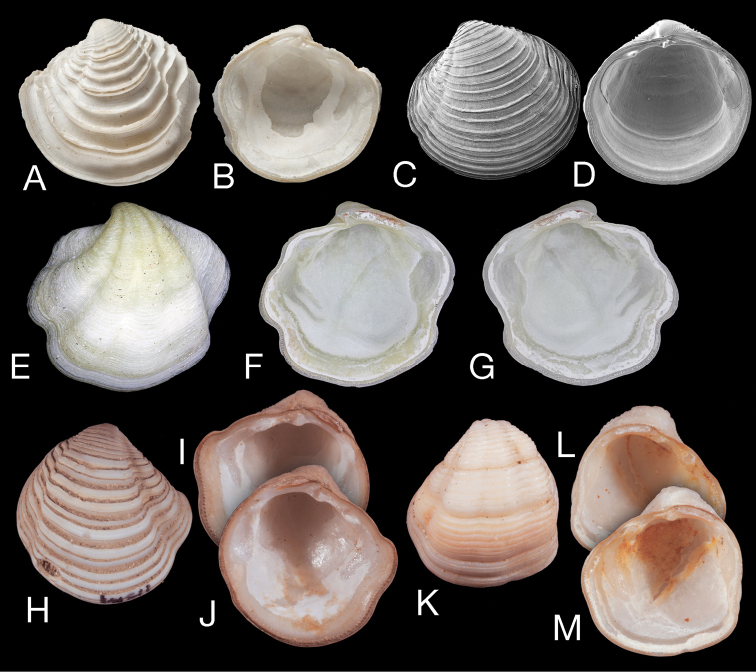
Other *Pleurolucina* species. **A, B** *Pleurolucina hendersoni* Britton, 1972, exterior and interior of left valve Guadeloupe station GD 69 (MNHN), L 9.1 mm **C, D** *Pleurolucina sombrerensis* (Dall, 1886) exterior of left valve (L 4.9 mm) and interior of right valve (L 5.2 mm), USNM 446178, Eolis stn 48, off Miami, Florida, 110 m **E–G** *Pleurolucina undata* (Carpenter, 1865) exterior of left valve and interiors of right and left valves, NUMUK 1915.15.273 ‘California’, L 11.0 mm **H–J** *Pleurolucina leucocymoides* (Lowe, 1935), exterior of right valve and interiors of right and left valves SBMNH 141511, Baja California, NE of Isla Danzante, Mexico, L 11 mm **K–M** *Pleurolucina taylori* Coan & Valentich-Scott, 2012, holotype, exterior of left valve and interior of left and right valves, SBMNH 149647, Baja California, Los Frailes, Mexico, L 9.5 mm.

*Pleurolucina leucocyma* (Fig. 7) was thought to be widespread across the tropical Western Atlantic but we now consider it to be restricted to Florida and the Gulf of Mexico with the southern Caribbean records representing *Pleurolucina harperae*. The new species differs from *Pleurolucina leucocyma* (mean L 6.2 mm, H 7.4 mm, H/L 1.13) in being larger, less inflated and usually longer than high in the adult (Fig. 8). The radial folds are usually lower and the anterior adductor muscle scar is shorter and more divergent from the pallial line (Fig. 2 B). In shape and sculpture, it is most similar to the somewhat larger *Pleurolucina undata* (Figs 9 E-G) (mean L 15.1 mm, H 15 mm, H/L 0.95) from the eastern Pacific, Gulf of California, intertidal zone to 60 m (Coan and Valentich-Scott 2012).

Other less similar species are: *Pleurolucina hendersoni* (Figs 9 A, B) an offshore to deep water species (to 300 m) from the southern Caribbean (Cuba, Lesser Antilles) that reaches about 12 mm in length and resembles the eastern Pacific *Pleurolucina leucocymoides*. Compared with other *Pleurolucina*, the sculpture of broad radial folds is less pronounced and the commarginal lamellae are widely spaced and prominent. *Pleurolucina sombrerensis* (Figs 9 C, D) lives in deeper water to 200 m from the Florida Keys to Brazil. The shell reaches about 6–7 mm in length and is rounded in outline, with a shallow radial anterior sulcus and prominent close commarginal lamellae, sometimes separated by deep interspaces. It does not closely resemble other *Pleurolucina* but shares some shell features including dentition and adductor scar shape. The larger *Pleurolucina leucocymoides* (Figs 9 H–J) is known from shallow water to 150 m and ranges from Baja California to Ecuador and Galapagos Islands. The sculpture of broad prominent commarginal lamellae and absence of prominent radial folds distinguish it from other *Pleurolucina*. Lastly, *Pleurolucina taylori* (Figs 9 K–M) is known from the intertidal zone to 183 m in the Gulf of California; it is distinguished by the highly inflated shell and closely spaced, low commarginal lamellae with four to five radial folds and resembles the extinct late Pliocene – mid-Pleistocene Floridian species *Pleurolucina amabilis*.

## Discussion

*Pleurolucina* is a genus of seven living species from the tropical to subtropical western Atlantic and eastern Pacific with none recognised from the eastern Atlantic or Indo-West Pacific. In that respect, it is similar to *Radiolucina* (Garfinkle 2012) and *Lucinisca* that share similar distributions. In the western Atlantic, the most similar species to the southern Caribbean *Pleurolucina harperae* is *Pleurolucina leucocyma* from Gulf of Mexico and Florida. This distributional pattern of northern and southern species pairs is seen in *Ctena* (*Ctena orbiculata* and *Ctena imbricatula*), *Lucinisca* (*Lucinisca nassula* and *Lucinisca muricata*) and *Lucina* (*Lucina pensylvanica* and *Lucina roquesana*) (see Taylor and Glover submitted). Cognate pairs of bivalves have been recognised from morphology and/or molecules on either side of the central American Isthmus (Marko 2002, Marko and Moran 2009). Although molecular confirmation is lacking, *Pleurolucina harperae* is similar in shell form to *Pleurolucina undata*, *Pleurolucina hendersoni* resembles *Pleurolucina leucocymoides* and perhaps *Pleurolucina leucocyma* is a sister species to *Pleurolucina taylori*.

An interesting and unusual feature of *Pleurolucina harperae* is the repeated conchiolin sheets that are calcified with layers of embedded spherules. A model of conchiolin sheet formation in another lucinid genus, *Cardiolucina*, was proposed by Ishikawa and Kase (2007 fig. 7). Periodically, normal shell secretion of outer, middle and inner shell layers stops and a conchiolin sheet is secreted across the inside of the shell from the margin and extending within the pallial line. This break in normal calcification is marked by a distinct notch at the shell surface. Calcification then resumes with secretion of normal shell layers. Conchiolin layer formation in *Pleurolucina* is essentially similar but each layer is thicker with repeated sublayers of aragonitic spherules. The narrow channels linking successive spherule layers suggest some sort of original tissue connection to the cells of the mantle surface.

Conchiolin layers within the shell have been recorded in several bivalve families but those in the Corbulidae have attracted most attention because of the supposed resistance to predation by drilling gastropods evidenced by the high incidence of failed borings that terminate at the organic layers (e.g. Lewy and Samtleben 1979, Harper 1994). Alternatively, organic layers may enhance resistance to shell dissolution, endolithic organisms or shell breakage (Anderson 1992, Harper 1994, Kardon 1998). In contrast to *Pleurolucina* where the conchiolin layers are secreted episodically, the layers in Corbulidae are secreted continuously as a sublayer of normal shell formation. In *Corbula gibba* the conchiolin layer is calcified with cone-shaped spherules approx. 8 µm in diameter (Lewy and Samtleben 1979 figs 5A–F). The organic layers of *Pleurolucina harperae* are similar in position and mode of formation to those recorded for species of *Cardiolucina* (Ishikawa and Kase 2007), but are much more highly calcified. *Cardiolucina* spp also show a high incidence of multiple drill holes with many terminating at the organic layers (Ishikawa and Kase 2007). *Pleurolucina* and *Cardiolucina* are not closely related among the Lucininae and the occurrence of conchiolin layers in other lucinids seems to be sporadic and certainly absent in many genera although no comprehensive study has been made. Nonetheless, calcified conchiolin layers do occur in some individuals of *Lucina pensylvanica* that is more closely related to *Pleurolucina*. It is tempting to regard the conchiolin layers as an adaptation conferring some resistance to shell drilling predation but, as argued in the case of *Corbula* (e.g. Kardon 1998), the layers may be an exaptation having first developed with some other function such as resistance to shell dissolution or enhancement of mechanical strength.

## Supplementary Material

XML Treatment for Pleurolucina

XML Treatment for Pleurolucina harperae
